# Pharmacologically-induced stress has minimal impact on judgement and attention biases in sheep

**DOI:** 10.1038/s41598-019-47691-7

**Published:** 2019-08-07

**Authors:** Jessica E. Monk, Sue Belson, Caroline Lee

**Affiliations:** 1 CSIRO, Agriculture and Food, Armidale, 2350 Australia; 2 University of New England, School of Environmental and Rural Science, Armidale, 2350 Australia; 3 Sheep CRC, University of New England, Armidale, 2350 Australia

**Keywords:** Psychology, Cognitive neuroscience, Animal behaviour, Animal physiology

## Abstract

The emotional impact of exposure to stressors has not been well quantified in animals. We hypothesised that exogenous induction of stress in sheep would induce a pessimistic judgement bias and increased attention towards a threatening stimulus, suggestive of a negative emotional state. Stress was induced pharmacologically by administering synthetic adrenocorticotropic hormone. Judgement bias was assessed using a spatial go/no-go task after exposure to acute stress (one injection), chronic stress (21 daily injections) and acute-on-chronic stress (2 min isolation after 28 daily injections). Attention bias was assessed during chronic stress only (22 daily injections). In contrast with our hypotheses, there was no strong evidence that Synacthen administration altered judgement bias or attention bias at any stage of the experiment. Stressed sheep were more likely to approach ambiguous locations than saline Control animals, however, statistical evidence for models fitting treatment group was very weak. Overall, our findings suggest that elevated levels of cortisol may not fully explain changes to judgement bias observed in previous studies after environmentally-induced stress. Further studies are required to better understand which aspects of environmentally-induced stress alter judgement bias and to further validate cognitive methods of assessing affect in sheep.

## Introduction

Domesticated animals regularly experience stressful events and environments which may impact their physical and mental well-being. However, the impact of stress on the affective (or emotional) states of animals is not fully understood. The stress response involves activation of the hypothalamic-pituitary-adrenal (HPA) axis, which leads to a series of highly conserved neuroendocrine reactions allowing animals to physiologically, behaviourally and psychologically respond to homeostatic threats^1,2^. This response is typically considered to be adaptive, however when a stressor is prolonged or repeated, the stress can become chronic and may lead to dysregulation of the HPA-axis and maladaptive stress responses^3,4^. In humans, this can lead to mood changes and has been implicated in the development and maintenance of depressive and anxious emotional states^3,5^. In addition to the impact of chronic stress itself, an increased effort to maintain homeostasis (allostatic load) can reduce the ability of animals to cope with and adapt to additional acute stressors, and may also lead to further shifts in affective state^4,6–10^. Thus, the potential impact of stress, particularly chronic stress, on the mental well-being of animals and their ability to cope with additional stressors deserves further attention.

A key limitation for the study of affective states in animals has been a lack of quantitative assessment methods. To address this limitation, an increasing body of literature has assessed emotional states via their impact on an individual’s cognitive processing of information, termed cognitive bias. Many of these studies have focused on sheep, which are farmed globally in large numbers and are used in biomedical research^11–14^. The type of cognitive bias most widely studied in sheep is judgement bias, where affective state can alter an individual’s interpretation of ambiguous situations^15,16^. Individuals in positive affective states typically make more positive judgements about ambiguous information (optimism), while individuals in negative affective states typically make more negative judgements about ambiguous information (pessimism)^14^. Judgement biases can be assessed using a range of test paradigms. For example, in a go/no-go spatial discrimination task, subjects are trained to discriminate two locations, such that they approach the rewarded location (go) and avoid the negative or non-rewarded location (no-go). Subjects are then presented with an ambiguous, intermediate location and their go or no-go response is interpreted as the subject perceiving the ambiguous cue to be positive or negative respectively. In sheep, animals treated with an anxiolytic drug or an opioid, to induce positive affective states, were more likely to approach ambiguous locations during a go/no-go spatial discrimination task, suggesting they were more optimistic^17,18^. Sheep treated with a serotonin inhibitor to induce a depressed state were less likely to approach ambiguous locations^19^. These studies show that judgement bias tests may be used as a measure of affective states in sheep.

Another type of cognitive bias which has been more recently studied in animals is an attention bias, where affective state can alter an individual’s allocation of attention towards different types of information^20^. For example, humans in anxious states pay more attention towards threatening information than non-anxious individuals^21–23^. Tests for attention bias are potentially a more rapid and practical option for assessing affective states in animals, compared to typical judgment bias test paradigms. In sheep and cattle, a novel attention bias test has been developed and pharmacologically validated as a potential measure of anxiety-like states, where animals given an anxiogenic drug spent more time looking towards a threatening stimulus and displayed increased vigilance behaviour compared to control animals^24–26^. Further, behavioural responses in a modified version of the test have also been shown to reflect pharmacologically-induced depression-like states in sheep^27^. Together, these studies show that attention bias tests can also potentially be used to assess affective states in sheep. Examination of judgement and attention biases together may provide additional information on the affective states of animals. Recently, studies in humans have also highlighted a need to better understand the way in which these biases interact and relate to one another, for a greater understanding of their underlying mechanisms^28^. Examining the relationships between cognitive bias tests in animals may allow us to gain a greater understanding of the mechanisms driving cognitive biases, which may include stress response pathways.

A number of studies in sheep have examined the impacts of environmental manipulation to induce endogenous acute and chronic stress on cognitive biases, however the results have not always been consistent between studies. Using a go/no-go spatial discrimination judgement bias task, pessimistic judgement biases were observed in sheep after 3 weeks^29^ and 9 weeks^30^ of unpredictable, uncontrollable exposure to aversive husbandry procedures, expected to have induced chronic stress. Two further studies found several months of unpredictable, stimulus-poor housing only had a weak effect on judgement bias, with one study finding a slight pessimistic bias^31^, while the other found a slight optimistic bias^32^. In contrast, three days of restraint and isolation stress induced an optimistic bias in sheep^33^, as did an acute shearing challenge^34^. Most recently, Verbeek *et al*.^35^ examined the impact of chronic stress induced by 9 days of lying deprivation on both judgement and attention bias in sheep. Further, they examined the impact of an additional acute stressor (shearing) on judgement bias. Prior to the shearing challenge, chronically stressed sheep appeared to be more optimistic during judgement bias testing and showed an attention bias away from a threat when compared to control animals, consistent with a more positive emotional state. After shearing, no differences in optimism were evident between the groups. It has been proposed that the unexpected optimistic judgement biases observed in many of these studies may reflect a positive emotional state caused by release from the imposed environmental stressors. This highlights a key challenge in using environmental manipulations to alter the state of an animal, as it can be difficult to identify which state the manipulation has induced and to maintain the state throughout the testing period^33,34^.

Pharmacological treatments can alter the state of an animal in a more standardised and repeatable manner than environmental manipulations, while remaining active for the duration of testing^36,37^. Further, pharmacological treatments can be readily matched with appropriate controls (e.g. saline injections). A number of studies have examined the impact of pharmacological treatments on cognitive biases in animals, with most pharmacological agents aiming to alter the affective states of the subjects^19,24,27,38,39^. Few studies have examined the impact of exogenous induction of stress on the affective states of animals through pharmacological manipulation. The link between stress and affective states is well established in humans and the mechanism of action is well described, with exogenous glucocorticoids shown to readily cross the blood-brain barrier to access the emotion related parts of the brain, including the amygdala. Adrenergic receptors have been found in the amygdala and play an important role in fear processing and memory for emotionally relevant information^40^. In addition, the link between mood disturbances, such as psychosis, and glucocorticoid administration has been confirmed in humans^41^. In animals, two studies have investigated the impact of pharmacologically-induced stress on judgement biases. In rats, injection of corticosterone and a noradrenaline reuptake inhibitor, to mimic the early stages of the acute stress response, caused a pessimistic bias in an audio discrimination judgement bias task^42^. In chickens, seven days of corticosterone administration, expected to induce chronic stress, caused a pessimistic bias during a spatial discrimination judgement bias test^43^. Conducting a similar study in sheep, stimulating the HPA axis via exogenous induction of stress, could help to better understand the impact of stress on cognitive biases and affective state. Previous studies in sheep have developed a model using daily injections of Synacthen, a synthetic adrenocorticotropic hormone, over a period of 4 weeks to chronically elevate plasma cortisol concentrations and induce a stress response^44,45^. Synacthen may therefore be a suitable candidate drug to pharmacologically validate the effect of acute and chronic stress on the affective states of sheep.

The aim of the current experiment was to determine the impact of stress on the emotional states of sheep, examining the effects of acute, chronic and acute-on-chronic stress. The study followed a similar design to Verbeek *et al*.^35^, assessing emotional state using judgement bias and attention bias tests, however acute and chronic stress states were induced pharmacologically using Synacthen, instead of using environmental manipulations. We hypothesised that sheep given one injection of Synacthen (Stress; acute stress stage) would be more pessimistic than control animals given one saline injection (Control; acute stress stage) in a judgement bias test. Further, we hypothesised that continued daily injections of Synacthen over a 3 week period (Stress; chronic stress stage) would induce a more pessimistic judgement bias and an increased attention bias towards a threat compared to animals given daily saline injections (Control; chronic stress stage). Preliminary analyses were conducted to compare animal responses in the judgement bias and attention bias tests on days 21 and 22. It was expected that more pessimistic sheep would show increased vigilance and attention to the dog, consistent with a negative emotional state. Finally, we hypothesised that an additional acute stressor (2 min of isolation) would induce a more pessimistic judgement bias in the chronically stressed animals compared to the control animals exposed to an acute stressor only (acute-on-chronic stage). It was expected that the Stress group would become increasingly more pessimistic between the acute, chronic and acute-on-chronic stress stages. Plasma cortisol concentrations were assessed during the experiment to confirm the effect of Synacthen administration on HPA-axis responses.

## Results

### Judgement bias

One animal from the Stress group failed to go to the Positive (P) location on day 1 of judgement bias testing, but approached the P location on the other test days. Data for that sheep were retained for analyses. The other 26 sheep approached the P location on all test days.

The maximal model fitted to the judgement bias data included fixed effects of location, treatment, test day and all interactions. A subset of the best ranked models from the “dredge” function in R (Δ*i* < 4) are presented in Table 1. The null model consisting of the intercept only had a high Δ*i* AICc of 173 and Δ*i* BIC of 157.

**Table 1:** Factors influencing animal responses during judgement bias testing.

Model	df	Δ*i* BIC	*w*_*i*_ BIC	Δ*i* AICc	*w*_*i*_ AICc
location	7	**0**	**0**.**76**	8.71	0.01
location + treatment	8	2.37	0.23	7.16	0.02
location * treatment	12	10.79	0.0	**0**	**0**.**77**
location * treatment + day	14	21.39	0.0	2.87	0.18

Estimated information criterion were obtained using the “dredge” function in R, where the maximum generalised linear mixed effects model included fixed effects of treatment, location, day and all interaction terms. Random effects were day nested within animal ID for each model. Models are listed in order of increasing Δ*i* BIC. Only models with a Δ*i* BIC or Δ*i* AICc < 4 are presented. The highest ranked models are emphasized with bold font.

Δ*i*; differences in information criterion values compared to the optimal model (lowest BIC or AICc) within the given set of models. w*i* BIC and w*i* AICc: Schwarz and Akaike weights respectively, indicates relative weight of evidence for each model within the given set of models.

**Figure 1:**
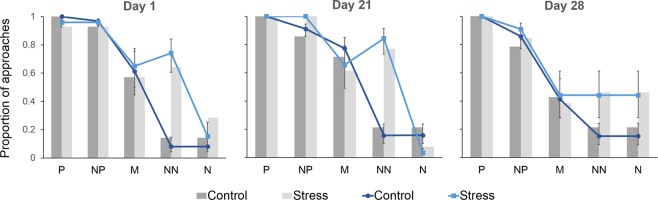
Mean predicted values ± 95% CI from a generalised linear mixed effects model including fixed effects of treat, location, day and all interaction terms during judgement bias testing. Test day nested in animal ID was included as a random effect. The bar charts on each panel show the total number of go responses as a proportion of the number of entries into the judgement bias test for each location on the given test day (P, positive location; NP, near positive, M, middle; NN, near negative; N negative location).

All models listed in Table 1 included location as a fixed effect. The best-ranked models by AICc also included treatment as a fixed effect, however support for a treatment effect was considerably weaker when considering BIC (Table 1). Model predictions from the maximum model are given in Fig. 1. Predicted values from this model suggest that sheep from the Stress group were more likely to approach the ambiguous locations than Control animals. This is particularly demonstrated by the observation that the Stress group were approximately 3 times more likely to approach the Near-Negative (NN) location than the Control group across all test days (raw proportion of combined go responses 0.63 and 0.19 respectively, Fig. 1). Evidence that test day had an effect on approach was negligible. One of the models fitting day as a fixed effect had a Δ*i* AICc < 4, however the Δ*i* BIC value for this model was greater than 20 (Table 1).

### Attention bias

**Figure 2:**
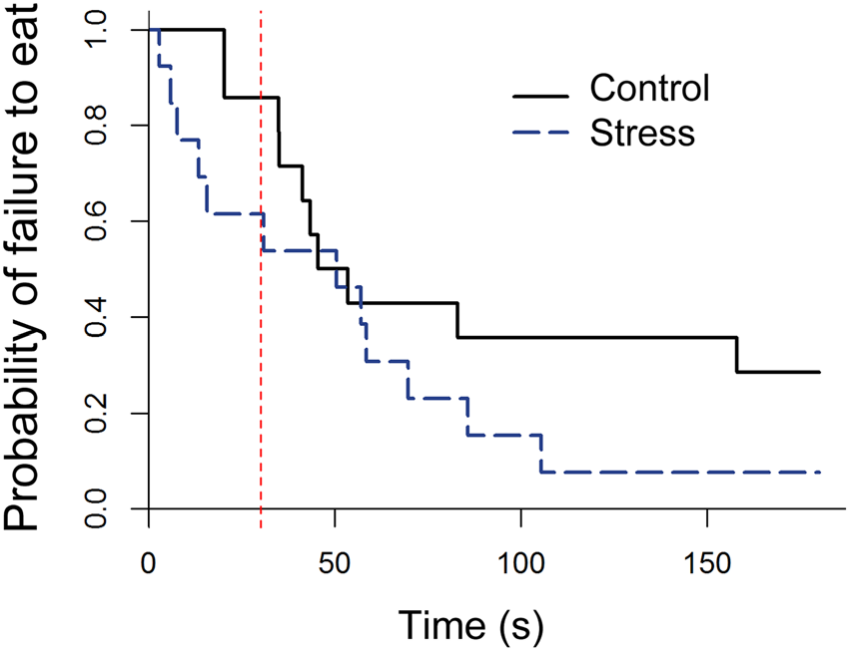
Kaplan-meier curves for latency to eat in the attention bias test for the model fitting treatment group. The red dotted line indicates the time at which the dog window was covered during testing. For each time an animal ate the food, the probability on the Y axis drops.

Control animals had a greater latency to eat than Stress animals (89.6 and 52.5 s respectively), however this difference was not significant in the Cox proportional hazards model (Log likelihood ratio (1) = 1.83, P = 0.2; Fig. 2). All other variables examined were best described by a model fitting the intercept only, as opposed to models fitting treatment group (Table 2). For amount of food eaten and time spent immobile, models fitting treatment group also had some evidence.

**Table 2:** Predicted means and estimated information criterion for behaviors in the attention bias test.

Variable	Predicted means [95% CI]		Treatment model			
	Control	Stress	w*i* BIC	Δ*i BIC*	w*i* AICc	Δ*i AICc*
Attention to dog (s)	22.8 [14.8; 35.4]^&^	18.6 [11.8; 29.3]^&^	0.20	2.82	0.26	2.07
Vigilance (s)	123.1 [109.6; 136.5]	117.5 [103.5; 131.4]	0.19	2.92	0.25	2.16
Food eaten (g)^$^	70.9 [29.3; 112.4]	93.5 [50.4; 136.5]	0.21	2.65	**0**.**28**	**1**.**90**
Time immobile (s)	3.3 [1.0; 8.2]	1.4 [0.1; 4.2]	**0**.**27**	**1**.**95**	**0**.**35**	**1**.**20**
Zones crossed (*n*)	14.6 [9.2; 22.7]	14.7 [9.2; 23.2]	0.16	3.3	0.22	2.54

Parameters were obtained using the “dredge” function in R, comparing a generalised linear model fitting the intercept only (Int) with a model including Treatment as a fixed effect. Models with a Δ*i* < 2 are emphasized with bold font.

^&^predicted means and CI calculated on the log scale, back-transformed values are presented. ^$^variable was analysed for the entire test period (with and without dog), all other variables were analysed for the period without the dog present only. w*i* BIC and w*i* AICc: Schwarz and Akaike weights respectively, indicates relative weight of evidence for each model within the given set of models Δ*i*; differences in information criterion values compared to the optimal model (lowest BIC or AICc) within the given set of models

### Cortisol response

**Figure 3:**
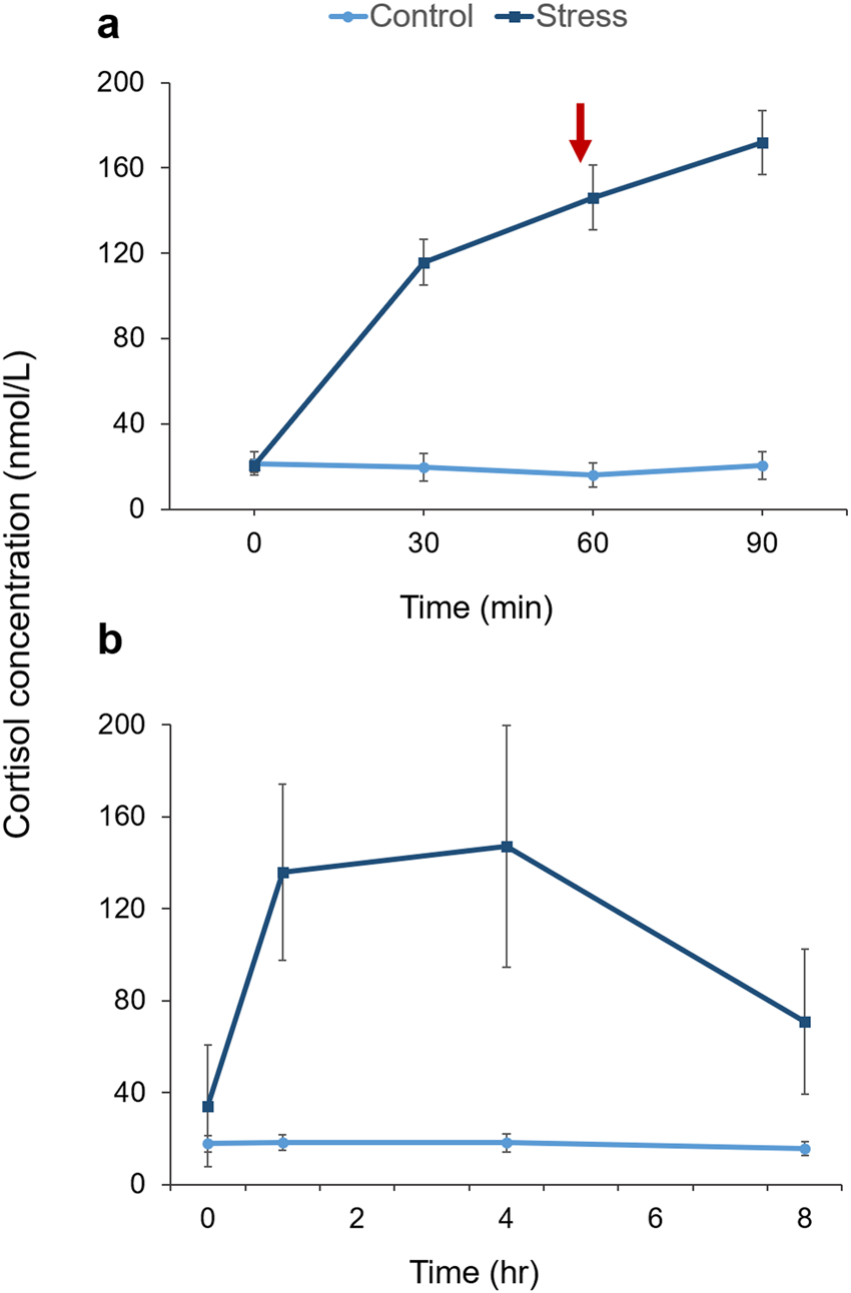
Cortisol response on days 1 (**a**) and 29 (**b**) of the trial after injection of Synacthen (Stress) or Saline (Control). Predicted means ± 95% CI from a linear mixed effects model are given for each timepoint. The baseline blood sample was taken immediately prior to injection (Time 0). The red arrow indicates time of judgement bias testing on day 1.

There was strong evidence for the model fitting timepoint, treatment and their interaction for cortisol response on day 1 (Table 3). For cortisol response on day 29, there was strong evidence for the model fitting treatment only (Table 3). Animals treated with Synacthen showed a greater cortisol response than Control animals on both days of testing (Fig. 3). Control animals showed little deviation from the baseline cortisol concentration on either test day (Fig. 3).

**Table 3:** Influence of time and treatment on cortisol responses after injection of Synacthen on days 1 and 29.

Day	Model	df	Δ*i* BIC	*w*_*i*_ BIC	Δ*i* AICc	*w*_*i*_ AICc
1	treat * time	7	**0**	**1**	**0**	**1**
	intercept	4	187.0	0	194.1	0
29	treat	5	**0**	**0**.**90**	**0**	**0**.**70**
	treat + time	6	4.66	0.09	2.22	0.23
	intercept	4	16.3	0	18.8	0

Estimated information criterion were obtained using the “dredge” function in R, where the maximum generalised linear mixed effects models included treatment, sample time and their interactions as fixed effects and sheep ID as a random effect. Models are listed in order of increasing AICc. Models with a Δ*i* < 2 are emphasized with bold font.

Δ*i*; differences in information criterion values compared to the optimal model (lowest BIC or AICc) within the given set of models. w*i* BIC and w*i* AICc: Schwarz and Akaike weights respectively, indicates relative weight of evidence for each model within the given set of models.

### Relationship between judgement and attention biases

**Figure 4:**
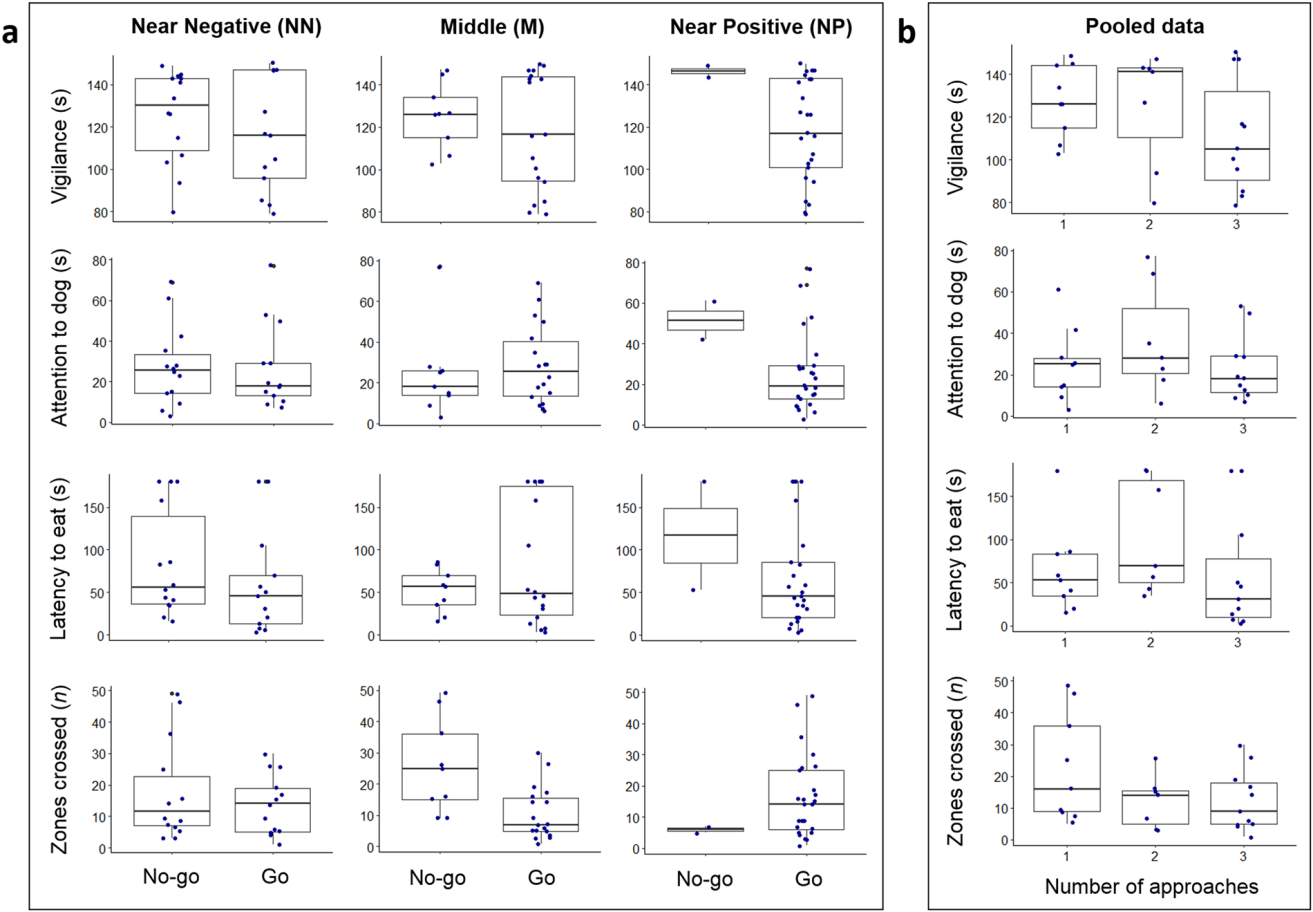
Boxplots displaying the distribution of behavioural responses observed during attention bias testing, grouped by the go/no-go responses to ambiguous locations (**a**) or the summed number of approaches (**b**) during judgement bias testing on day 21. Boxplots show the median durations or counts, the interquartile range (IQR) and the range of the data within 1.5 × IQR. The dots represent raw duration or count data for individual sheep. The number of animals which approached the NN, M and NP locations were 13, 18 and 25, respectively (**a**). The number of animals which approached one, two or three of these ambiguous locations were nine, seven and 11, respectively (**b**).

Figure 4 shows the raw number of go responses of sheep to ambiguous locations on day 21 of judgement bias testing, plotted against key behavioural responses during attention bias testing on day 22. Jonckheere-Terpstra tests showed no significant relationships between the number of go responses and behavioural responses during attention bias testing (P values ranging between 0.20 and 0.85).

## Discussion

In contrast with our hypotheses, administration of an exogenous glucocorticoid (Synacthen) to induce stress had no strong effect on judgement bias or attention bias in sheep, relative to the control animals. Further, the results do not support our hypothesis that the judgement biases of stressed sheep would change between the acute, chronic and acute-on-chronic stress stages. There was some evidence for an optimistic bias in the stress animals at all time points, which is consistent with some^33–35^, but not all previous studies^29,30,46^. However, statistical evidence for this optimistic bias was weak. The effect of Synacthen on the Stress animals was confirmed by an increased plasma cortisol concentration on days 1 and 29. This response is similar to that observed previously in sheep administered Synacthen daily for 4 weeks^44^, and provides physiological evidence that the induced cortisol responses persisted for the duration of judgement bias and attention bias testing. A lack of effect on cognitive bias therefore suggests that elevated levels of cortisol cannot fully explain changes to judgement biases observed in previous studies after environmentally-induced stress^35^. Alternatively, these results may suggest that the cognitive bias tests used in this study could not detect changes to affective state caused by increased circulating cortisol concentrations. Further studies are therefore required to better understand which aspects of environmentally-induced stress may be contributing to an altered judgement bias in sheep, and to further validate cognitive bias tests as measures of affective state.

It is possible that administration of exogenous glucocorticoids did not impact on the affective states of sheep. A lack of effect could be due to the use of an exogenous hormone to induce stress, which may not equate to the stress response caused by an environmental manipulation, that impacts on an array of neurophysiological pathways. Alternatively, it may suggest that glucocorticoids do not have a role in modulating affective state in sheep, regardless of their origin. Previously, environmentally-induced elevations in cortisol have been observed without alterations to judgement bias in pigs^47^, and altered judgement bias has been observed without evidence of changes to endogenous cortisol concentrations in sheep. However, studies in humans have established a clear link between administration of exogenous glucocorticoids and affective states^40^. Further, direct relationships between exogenous corticosteroids and judgement bias have been demonstrated in chickens and rats^42,43^. We therefore suggest our inability to replicate these results in sheep may be due to the limitations of the cognitive bias tests we used and our study design, as discussed below.

It is possible that the cognitive bias tests were unable to detect a change in affective state in the Stress group, due to the confounding effects of feeding motivation or sensitivity to reward, as discussed by Verbeek *et al*.^35^. Glucocorticoids have been shown to increase the salience of pleasurable and compulsive activities in humans and rodents, such as ingestion of sugars, fats and drugs of abuse^48–51^. Increased motivational behaviours for rewarding stimuli and ingestion of “comfort foods” are thought to reduce the negative consequences of stress by downregulating HPA-axis activity^52,53^. Further, it has been proposed that animals may seek positive or rewarding experiences to counteract negative experiences^54^. In sheep, cortisol responsiveness has been linked to metabolic and behavioural traits associated with obesity^55^. As such, an increased motivation to eat palatable food rewards in the Stress group may have masked a potential change in judgement bias and attention bias, and may explain the observation of a weak optimistic bias during the current study.

Another factor which may have limited the ability of the judgement bias test to detect a response is the presence of olfactory cues from the dog and food during testing. Ideally, testing of ambiguous locations would occur in the absence of any cues^56^, however this is often difficult to achieve in a practical setting. Instead, both positive and negative cues can be kept in place throughout all training and testing, as to not include an additional olfactory cue which may signal a particular outcome during ambiguous trials. In the current study however, the scent of the positive location changed between the positive and negative training sessions as, while some food was present next to the arena at all times, this scent may have been stronger during positive trials when there was food located inside the arena. This means the strength of the scent of food during the ambiguous trials was the same during negative training trials, potentially providing an additional cue that the outcome would be negative. If this were the case, we might expect a low proportion of go responses across all treatment groups to the ambiguous locations, however this was not observed, which suggests the presence of olfactory cues had minimal impact on results. Nevertheless, it cannot be ruled out that elevated cortisol levels enhanced the animals’ perception of the scent of the food outside of the arena during ambiguous trials, such that they were more likely to expect a positive outcome. Further studies should be aware of and control for this potential effect during judgement bias tests as described by Verbeek *et al*.^35^.

An effect of Synacthen on the Stress group may not have been detected if the Control animals had an equivalent stress response, due to the impact of the treatment and testing procedures. This is not supported by the cortisol responses of the Control animals, that remained very low on days 1 and 29 during judgement bias testing and blood sampling. Further, sheep often voluntarily moved into the testing areas during the experiment, being led by a familiar handler with a feed bucket. Therefore, it seems unlikely that the Control animals found the treatment and testing procedures to be highly aversive. Nevertheless, the judgement bias and attention bias testing procedures involved isolation, novelty and exposure to predators, which are innately stressful for sheep^57–59^. Thus, it cannot be ruled out that the treatment and testing procedures impacted on Control animal responses during cognitive bias testing, so that they could not be distinguished from the Stress group.

Regardless of an impact on affective state, it makes biological sense for an animal which has been recently threatened and that is undergoing a physiological stress response to be more vigilant and pay more attention towards additional threats^60,61^. A lack of response during attention bias testing in the chronically stressed animals may therefore suggest that the dog and novel environment during attention bias testing were not perceived to be threatening and therefore did not elicit an additional response from the Stress group. Although as discussed previously, this seems unlikely given that isolation and predators are innately stressful for sheep. Another possibility is that the Stress group perceived the dog as a threat more strongly than Control animals, but failed to respond accordingly with an appropriate behavioural response, potentially demonstrating a maladaptive response associated with chronic stress and increased allostatic load^4,6–10^. Finally, a lack of response may again indicate that the pharmacological model of chronic stress was not effective as a manipulation of affective state, or that the attention bias test was not sensitive enough to detect an effect of the drug on the animals’ behaviour. Further studies examining the impact of stress on the attention bias test only, using naive animals, would provide further insight into these issues.

No clear relationships were observed between animal responses in the judgement and attention bias tests during the chronic stress stage of this study. This finding suggests that each test may provide different information on the affective state of an animal. It has been suggested that judgement biases are largely influenced by longer term mood states, while attention biases are more situation specific and may reflect shorter term emotional responses^62^. Nevertheless, recent studies in humans have demonstrated relationships between judgement and attention biases^63,64^. It is possible that no relationships between cognitive biases were observed in the current study due to limited data availability and low statistical power. Additional studies using larger numbers of animals will help to better understand the nature of the relationship between judgement and attention biases in animals, if one exists. Such studies may help to inform on the underlying mechanisms driving cognitive biases in animals.

It is important to consider the potential impact of repeated assessments on animal behaviour during testing, both between and within the two experiments^35^. The attention bias test is still relatively new and the effects of repeated testing on animal behaviour have not yet been explored. Therefore, exposure to the attention bias test during the previous study may have altered animal responses during the current study. Additionally, while a different dog was used in the attention bias test than for judgement bias training and testing, repeated exposure to dogs may have reduced the animals’ responses to this potential threat during the attention bias test. Another potential limitation of the study was that it only applied one test session at each stage of the experiment, with a single exposure to each ambiguous stimulus per session. As animal responses can vary considerably between trials due to day to day variation, this may have limited our ability to detect an actual effect in the study. On the other hand, there are also limitations to repeated judgement bias testing as animals learn the ambiguous cues are not rewarded upon repeated testing, and begin to approach ambiguous cues less often^65–68^. In the current study, we observed no effect of day on judgement bias test responses. Further, the study design was balanced so that each treatment underwent the same number of tests. As such, we do not expect repeated testing within the current study to have affected the differences in judgement bias between treatment groups.

Animals allocated to a stress treatment during the previous study^35^ were balanced between the Control and Stress groups in the current study, however, it is important to acknowledge the potential effect of earlier stress-inducing treatments on animal responses during the current study. Early life stress or neglect has been shown to impact judgement biases later in life in rats and female goats^69–71^. Even prenatal stress has been found to influence judgement bias in lambs^72^. Thus, it would not be surprising if the treatments applied to animals in the previous study impacted their responses in the current study. However, due to the small number of animals used in the current study, we were unable to further investigate these potential effects. Using the same sheep between studies reduced the time taken for animals to reach the necessary learning criteria for judgement bias testing. However, due to the potentially confounding effect of previous experiments on animal responses, through earlier treatments and habituation to the test procedures, it is suggested that future studies use naive animals for cognitive bias testing, unless it relates to the hypotheses being tested.

It has been proposed that the differences in results between previous judgement bias studies may be due to the duration of the applied stressors, where chronic stress has more consistently caused a shift towards pessimism, while acute stress has yielded mixed results^14,73^. This theory is not supported by the findings of the current study, which found no evidence of a pessimistic judgement bias after chronic stress, or an optimistic bias after acute stress. Further, this is not supported by the results of Verbeek *et al*.^35^, which observed an optimistic bias after chronic stress. However, it should be noted that the durations over which chronic stress have been induced vary greatly between studies^29–32,35^, and that studies intending to induce chronic stress may not have done so successfully. Alternatively, it has been suggested that previous judgement bias studies may have incorrectly inferred the affective states induced by the given treatments, by failing to consider mismatches between the animals’ perceived expectations and outcomes during affective state induction or testing^74^. However, this theory is also unsupported by the current study, as well as a recent meta-analysis which aimed to determine whether such mismatches could predict judgement bias test outcomes more accurately than the inferred affective states^75^.

The differing effects of stress on judgement bias responses in previous studies could also potentially be explained by differences in assessment methods or the breeds of sheep used in each study. Most studies in sheep have used a spatial discrimination task to assess judgement bias, however the types of positive and negative reinforcers have varied. Each of the studies which found an optimistic bias in stressed animals tested Merino sheep using a dog to reinforce the negative location^33–35^. Studies which found a pessimistic bias tested Romane sheep and used an air blower to reinforce the negative location^29,30,46^. Two further studies which found no effect on judgement bias tested Lacaune sheep using either an air blower^31^ or presentation of straw instead of feed^32^ as a negative reinforcer. A range of fear studies have observed differences in behavioural reactivity between sheep breeds^57,58,76^. Further, sheep have been shown to exhibit context specific patterns of behaviour, depending on the fear-eliciting stimuli presented^57,58,76^. Together these findings suggest the type of reinforcer and/or breed of sheep used in the experiments may have impacted the direction of the bias observed during previous judgement bias studies. Further studies aiming to examine the differences in judgement biases between sheep breeds or comparing different types of positive and negative reinforcers may shed light on the discrepancies observed between studies.

Overall, the results from this study do not support our hypotheses that pharmacologically-induced acute and chronic stress would cause pessimism and an attention bias towards threatening information in sheep. Further, our results show that increased circulating cortisol concentrations may not fully explain the responses observed during previous chronic stress studies, although the observation of a weak optimistic judgement bias is consistent with some previous results^33,35^. We suggest these findings should not be taken to say that chronic stress does not negatively impact on animal affective states. Instead, we suggest researchers should be cautious in their interpretation of cognitive bias test results in sheep until the methods are further validated. The study of affective states in livestock is a field of research in its infancy. Contradicting results between studies of judgement bias in particular show that tests for affective state require continued refinement and validation, before they can be considered robust measures of affect as an aspect of animal welfare.

## Methods

### Ethical statement

The protocol and conduct of this study were approved by the CSIRO McMaster Laboratory Animal Ethics Committee (Animal research authority #12–30), under the New South Wales Animal Research Act 1985. The protocol was carried out in accordance with all relevant guidelines and regulations. All animals were closely monitored for health and welfare during and after the experiments.

### Animals and management

Thirty-two maiden Merino ewes (18–19 months of age, average bodyweight 50.8 ± 3.9 kg) were used in this experiment, which was conducted in 2013 between April and May. All ewes were born on the same experimental farm in Armidale, Australia and were reared as one group after weaning. During training and testing, animals were grazed on pasture located approximately 50 m away from the handling and testing facilities. Sheep were walked between the pasture and testing facilities by a familiar handler. Sheep were fed a familiar supplementary ration of pelleted, lucerne-based concentrate each afternoon following training.

All of the ewes in the current study had previous experience with judgement bias and attention bias testing during the experiment conducted by Verbeek *et al*.^35^. Sheep were returned to the farm flock between the previous and current studies, for a period of 10 months, during which the sheep were managed as per normal farm practices. Readers are asked to refer to Verbeek *et al*.^35^ for further details of the previous experiment. Briefly, in the earlier study all ewes were trained to judgement bias testing over a 2 month period, then 30 of the sheep were randomly allocated to control and chronic stress treatment groups (*n* = 15 per group). The chronic stress group was subjected to social isolation and 18hrs of lying deprivation per day over 9 days, while the control animals were housed as a group in a paddock over the same time period. On day 9, all sheep were exposed to an additional acute shearing challenge. All sheep underwent judgement bias testing twice during the experimental period, before and after shearing. All sheep underwent attention bias testing once prior to shearing.

### Experimental design

A summary of the experimental protocol and timeline is given in Table 4. Details of each procedure are given in the following sections. As a general overview, sheep were retrained over a period of one month to undertake a judgement bias test. Twenty-eight sheep which successfully reached the training criteria were then pseudo-randomly split into two groups; Stress and Control treatments, balancing for positive location side and cue colour, as well as treatment allocation in Verbeek *et al*.^35^ (see below for details). All sheep underwent once daily injections from days 1 to 29 with saline (Control) or synthetic ACTH (Stress). On day 1, the first injection was expected to induce an acute stress state in the Stress group. By days 21 and 22, ongoing injections were expected to have induced a chronic stress state in the Stress group. On day 28, all animals underwent 2 min of isolation to induce acute stress in the Control group and acute on top of chronic stress (acute-on-chronic) in the Stress group. Judgement bias was assessed at each of these stages. Attention bias was assessed during the chronic stress stage as an additional assessment of affective state. Plasma cortisol response to the injection was assessed on days 1 and 29 of the experiment. Due to unforeseen circumstances, one animal in the Stress treatment group was removed from the study due to injury between days 1 and 21. Data from this animal were removed from the study. Only data for the remaining 27 animals were analysed.

**Table 4:** Experimental design and timeline.

Day	Procedure	Stage of experiment
−20	Begin daily judgement bias training	Training
0	Complete judgement bias training	
1	Begin once daily injections	Acute stress
	Judgement bias test 1	
	Blood sampling	
21	Judgement bias test 2	Chronic stress
22	Attention bias test	
28	Final daily injection	Acute-on-chronic stress
	Isolation box (2 min)	
	Judgement bias test 3	
29	Blood sampling	Blood sampling

### Judgement bias arena

**Figure 5:**
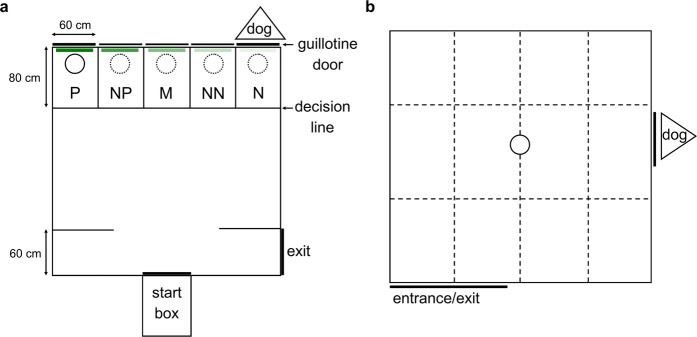
Schematic diagrams of the judgement bias (**a**) and attention bias (**b**) test arenas. The judgement bias test arena (**a**) had five potential bucket and cue locations; (positive (P), near positive (NP), middle (M), near negative (NN) and negative (N)). Only one location was accessible with only one bucket and coloured cue card present at a time during training and testing. The circles indicate the potential positions of a bucket which either contained feed (solid line) or was empty (broken lines). The diagram depicts the set up for sheep assigned to a Positive location on the left with a 95% colour density. The diagram was adapted from Verbeek *et al*.^35^. For a photograph of the arena, see Verbeek *et al*.^35^, however note that the bucket locations differed between studies. The attention bias arena (**b**) contained a familiar bowl holding feed (circle). A dog was visible through a small window for the first 30 s of the test, then the window was covered and dog was removed.

The current study used the same judgement bias arena described by Verbeek *et al*.^35^, adapted from Verbeek *et al*.^77^. Testing occurred in a 3 × 3 m arena surrounded by 1.5 m high wooden walls (Fig. 5). Sheep entered the arena through a start box centred along the front wall of the arena and exited through a door on the right (Fig. 5). The back wall of the arena was divided into five sections with wood panels 40 cm high (hereafter locations). Removable panels were located at the front of each location. A single location was made accessible to the test sheep for each trial or training session, by removing one of the front panels prior to testing. Sliding vertical doors were located behind the two most outer locations, which could be lifted to reveal a dog, lying down quietly (negative reinforcer). Dummy sliding doors identical in appearance to the actual doors were placed behind the three middle locations (ambiguous locations).

To facilitate discrimination between locations, five green coloured cue cards which differed in colour density (0, 25, 50, 75 and 95%) were created by adjusting the transparency of the same colour green in Microsoft PowerPoint^77,78^. The cues, printed on A3 paper and laminated, could be attached to the doors at each location in the judgement bias test. Therefore, a total of five cues and locations were used; positive (P, location of a bucket containing feed), near positive (NP), middle (M), near negative (NN) and negative (N, location of the dog). Only one location was accessible (front panel removed), with only one corresponding cue card present at a time during training and testing. Only the accessible location contained a bucket, which was placed in front of the closed sliding door, visible to the sheep. The bucket either contained a small feed reward (approximately 100 g of pelleted lucerne-based concentrate, P location) or was empty (all other locations). The bucket was located inside the arena during the current study, while the previous study positioned the bucket outside the arena behind the vertical sliding door at the P location^35^. This change in bucket location was made to better facilitate learning, by providing an additional visual cue for the location being tested.

### Judgement bias training

For two days before beginning judgement bias training, sheep were moved into the arena in groups of two for 5 min to re-habituate to the arena, with no buckets or cue cards present. Sheep then underwent one judgement bias training session per weekday for 4 consecutive weeks, following the procedure described by Verbeek *et al*.^35^. During retraining, each sheep was assigned the same Positive location (either the outer left or outer right location in the arena) and Positive cue card (0 or 95% colour density) assigned to them in the previous study. Sheep were first trained to approach their assigned P location, containing a bucket with a small feed reward, while all other locations were inaccessible. Once a sheep left the start box, they were given 3 min to step over the P location decision line with both front feet (go response) and begin consuming feed (Fig. 5). The decision line was physically visible to the sheep. The sheep were let out of the arena after consuming the feed, or if 3 min had elapsed without the sheep crossing the decision line (no-go response). Positive training was conducted over the first 2 training sessions. During the first session, sheep entered the test arena 3 consecutive times. During the second session, and for all subsequent training and testing days, sheep entered the test arena 5 consecutive times per session.

During the remaining training sessions, sheep were exposed to the P location for 3 of the arena entries and the N location for 2 of the entries in each training session. The sequences in which P and N locations were presented were randomised between sessions. During training to the N location, an empty bucket was placed in the corner location opposite the assigned P location (right or left) with an alternate cue card (95 or 0% colour density). If a sheep crossed the N location decision line (go response), the sliding door was opened to reveal a live dog sitting quietly. Once the sheep retreated from the bucket, the door was lowered and the sheep was let out of the arena. If the sheep did not approach a location within 30 s of exiting the starting box, the sheep was let out of the arena.

A sheep was considered to be trained when it correctly responded to 14 out of 15 consecutive positive locations and to 8 out of 10 consecutive negative locations. A ‘positive response’ was considered correct when the sheep stepped over the P decision line (go response) within 10 s of exiting the starting box. A ‘negative response’ was considered to be correct when the sheep did not cross the N decision line within 30 s of exiting the starting box (no-go response). During training, 4 sheep failed to reach these criterion and were removed from the study.

### Pharmacological treatments

All animals received an intramuscular (i.m.) injection once daily for 29 consecutive days (Table 4). Control animals were administered 0.5 ml of BP Saline while Stress animals were administered 0.5 ml of Synacthen Depot (0.5 mg of tetracosactrin zinc phosphate complex, Mallinckrodt Pharmaceuticals, UK). Synacthen has been used in sheep previously at this dose rate to chronically elevate plasma cortisol concentrations, mimicking the physiological response of the HPA axis to stress^44^.

Injections for each animal were staggered by approximately 10 min on days 1, 21, 22 and 28 to allow for judgement bias and attention bias testing, beginning and ending at approximately 8:00 AM and 1:00 PM respectively. On all other days, injections were administered to all animals at 8:30 AM. The injection site was rotated each day between the rump, thigh and dorsal muscles on the left and then right sides of the animals.

### Blood sampling and analysis

Blood sampling occurred on days 1 and 29 for assessment of plasma cortisol concentration as an indicator of HPA axis response. Blood samples were collected via jugular venepuncture into heparinised vacutainers on each of the sampling days at four consecutive time points. On day 1 (acute stress), samples were taken at time 0 (baseline prior to Synacthen injection) then at 0.5, 1 and 1.5 hrs post injection to evaluate the acute cortisol response to the Synacthen injection. Judgement bias testing occurred immediately prior to collection of the 1 hr post injection blood sample. On day 29 (chronic stress) samples were taken at time 0 (baseline prior to Synacthen injection) then at 1, 4 and 8 hrs post injection to evaluate the cortisol return to baseline.

Blood tubes were kept on ice until they could be processed. The blood samples were centrifuged at 2000 × *g* for 15 min at 5 °C, then the plasma was distributed into 2 ml aliquots that were stored at −80 °C until analysis for cortisol concentration. Plasma cortisol concentrations were measured using a commercial radioimmunoassay (Orion Diagnostica, Espo, Finland), previously validated for ovine plasma cortisol^79^. The intra-assay and inter-assay coefficients of variance (CV) for quality controls containing 24.9, 51.6 and 104.9 nmol/L of cortisol were 3.4, 5.7 and 7.7% and 6.5, 4.5 and 12.6% respectively.

### Judgement bias assessment

Judgement bias was assessed 3 times during the experiment, on days 1, 21 and 28, as described by Verbeek *et al*.^35^. Testing occurred 1 hr post injection for each given day, where treatment groups were spread evenly throughout the day. During testing, each sheep was released into the arena five consecutive times with each of the 5 locations presented once. The trained locations were reinforced with food (P) or exposure to the dog (N), however the ambiguous locations were not reinforced. Presentation of the locations occurred in one of the following orders: P, NP, M, NN, N (P first) or N, NN, M, NP, P (N first). Half the animals were pseudo randomly assigned to each start location on each day of testing, balancing between treatment groups, so that presentation order for each animal may have differed between test days. After leaving the start box, sheep were given a maximum of 30 s to respond for each location before being let out of the arena. The go or no-go responses were recorded from a video screen in real time. All tests were continuously recorded by two video cameras and a 16 channel Digital Video Recorder.

Immediately prior to judgement bias testing on day 28, each sheep was moved into an isolation box for 2 min to induce acute isolation stress^80,81^. The isolation box consisted of a 1.5 × 0.5 × 2 m wooden box with an open roof covered by shade cloth.

### Attention bias assessment

Attention bias was assessed as described by Verbeek *et al*.^35^, using a similar method to that validated by Lee *et al*.^24^. The test was conducted in a 4 × 4 m arena surrounded by a 1.5 m high fence covered in opaque black cloth (Fig. 5). The concrete floor was divided into 12 rectangular zones (1 × 1.2 m) marked on the ground with white paint. A familiar bowl containing 200 g of concentrate pellets was placed in the centre of the arena. A small window (77 × 58 cm) was located on one wall behind which a dog was sitting quietly (different from the dog used in judgement bias training). Each sheep was tested individually for a total of 3 min. The dog was visible to the sheep for the first 30 s of testing, then the window was covered by a retractable opaque cover and the dog was removed for the remainder of the test. All tests were continuously recorded by a video camera.

The following behaviours were recorded in real time or from video footage; time spent looking towards the dog or covered window with binocular vision (attention to dog^24^), duration with the head at or above shoulder height (vigilance^24,82^), latency to eat the food, number of zones crossed with both front legs and duration spent immobile with no movements of the head or body for greater than 3 s. Latency to eat was recorded from the beginning of the test. All other behaviours were recorded separately for the durations with and without the dog present. Only data without the dog present are presented, as this is the period where responses are to a perceived threat, as opposed to an actual threat posed by the presence of a predator, and thus better reflect anxiety states assessed using attention bias^24,35^. Between animals, all uneaten food was removed and the bowl refilled with new pellets so that the amount of food eaten by each sheep could be calculated. The arena was also cleaned after eliminations to avoid the influence of odour cues on further subjects. Vocalisations, eliminations and foot stamps were also recorded, however these data were not further analysed due a low number of occurrences during the test.

### Statistical analysis

The data were analyzed using R version 3.5.1^83^. All model residuals were checked for normality and homoscedasticity using Shapiro Wilks test for normality and visual assessment of Q-Q and residuals vs. fitted values plots where appropriate.

An information criterion based approach was used for model selection to determine which models most appropriately fit the data within a given set of models^35,84,85^. A maximal model was fitted for each analysis, then the parameters for models containing each possible combination of the predictors and their interactions were calculated using the “dredge” function from the package “MuMIn”^86^. We examined both the Akaike Information criterion adjusted for small samples sizes (AICc) and the Bayesian Information criterion (BIC), which more heavily penalizes model complexity. Due to this heavier penalisation, models selected by BIC are simpler and emphasize the key predictors, whereas model selection by AICc is typically preferred for model predictions^87^. Thus, our interpretation of results relied more heavily on BIC, however AICc is also presented. Models were selected based on Δ*i* (AICc or BIC difference relative to the smallest AICc or BIC value in the given set of models) and *w*_*i*_ (Akaike or Schwarz weight), indicating the relative weight of evidence for model *i* being the best fit for the data within the given set of models. Where multiple models had a Δ*i* < 2, those models could be considered equally likely to be the best fit for the data, however models with a Δ*i* < 4 also have considerable empirical support^88^.

### Judgement bias testing

The go/no-go responses during judgement bias trials were analysed using generalized linear mixed effects models using the package “lme4”^89^, as described by Gygax *et al*.^85^. Fitted models included all go/no-go responses across the 3 test days. The maximal model included fixed effects of location, treatment, day and all possible interactions between those variables. Location was fitted as a factor rather than a continuous variable, due to evidence of a non-monotonous pattern in go responses across locations. The random effects for each model were day nested in animal (id) to account for repeated measures across test days.

### Attention bias testing

Latency to eat data were analysed using a Cox’s proportional hazards model using survival analysis, as described by Monk *et al*.^25,90,91^. Animals which failed to eat within 180 s were deemed as censored results. Attention to dog, vigilance, food eaten and time spent immobile were analysed using linear models. Zones crossed data were analysed using a negative binomial general linear model, due to evidence of over-dispersion in the data. The maximal model for attention bias data fitted treatment group as a fixed effect only.

### Cortisol response

Cortisol data were analysed using linear mixed effects models. The maximum models for each day of sample collection included treatment, timepoint and their interaction as fixed effects and sheep id as a random effect. Data from one animal in the Control group on day 1 and two animals from the Stress group on day 29 were removed from the analysis due to abnormally high baseline cortisol concentrations (>150 nmol/L). Thus, data for *n* = 26 and *n* = 25 animals were analysed for days 1 and 29 respectively.

### Relation between judgement and attention biases

Analyses were conducted to determine the relationship between go responses during judgement bias testing on day 21 and key behavioural responses during attention bias testing on day 22. Due to the low amount of data available, go responses to ambiguous bucket locations (NN, M and NP) were pooled for further analyses. The number of go responses to any of the three ambiguous locations were summed for each animal. All sheep approached at least one location. Nine, seven and 11 sheep approached one, two or three of the locations, respectively. Jonckheere-Terpstra tests were then used to determine whether vigilance, attention to the dog, latency to feed or zones crossed behaviours during attention bias testing differed as the number of approaches to ambiguous locations increased. The Jonckheere-Terpstra test is a non-parametric, trend-based test, used due to non-normality of the data. Permutation tests were used for P-value estimation, based on 5000 permutations, due to the presence of tied values in the attention bias data.

## Data Availability

The datasets generated during the current study are available in the CSIRO Data Access Portal (DAP) 10.25919/5c8725f046880.
